# Retrospective voting and party support at elections: credit and blame for government and opposition

**DOI:** 10.1080/17457289.2016.1243543

**Published:** 2016-10-26

**Authors:** Carolina Plescia, Sylvia Kritzinger

**Affiliations:** ^a^ Department of Government, University of Vienna, Vienna, Austria

## Abstract

Retrospective voting is arguably one of the most important mechanisms of representative democracy, and whether or not the public holds the government accountable for its policy performance has been extensively studied. In this paper, we test whether retrospective voting extends to parties in the opposition, that is whether and how parties’ past performance evaluations affect their vote, regardless of whether they were in government or in opposition. Taking advantage of a rich set of questions embedded in a representative German national elections panel, we update our knowledge on the retrospective voting mechanism by modeling retrospective voting at the party level. The findings indicate that the incumbent status is not the only criterion for retrospective voting, ultimately suggesting that both government and opposition parties can expect credit and blame for their conduct and this should provide some impetus for responsive performance of all parties.

## 1. Introduction

Whether or not legislators are judged on what has happened in the past has significant implications for democratic accountability. Accordingly, a substantial literature has investigated the policy foci and time horizons that guide voters’ decisions. Almost without exception, the literature on electoral accountability examines voters facing a binary choice between either supporting the incumbent or not (e.g. Fiorina 1981; Berry and Howell 2007), mostly focused on the economic performance of government (e.g. Paldam 1991; Powell and Whitten 1993; Anderson 2007). This focus stems from the notion that evaluations of policy-making are restricted to parties in government (Duch and Stevenson 2008). Clearly, however, other parties influence policy-making and, in many contexts, (e.g. federal systems and minority governments), this influence is substantial (Strøm 1990; Powell and Whitten 1993; Lijphart 1999). Therefore, voters’ beliefs should reflect this wider notion of responsibility at the party level (Duch and Stevenson 2008; Duch and Falcó-Gimeno 2014). So far, very little empirical work has examined whether and how the public holds their *representatives* – and not only the government – accountable for the country’s current state of affairs.

We employ this wider notion of performance evaluations and model retrospective voting at the party level. While recent studies have started focusing on each party in the coalition government instead of the incumbent coalition governments as a whole (e.g. van der Brug, van der Eijk, and Franklin 2007; Fisher and Hobolt 2010; Debus, Stegmaier, and Tosun 2014; Williams, Stegmaier, and Debus 2015), we go one step further. First, we examine how retrospective evaluations of all parties, regardless of whether they were in office or not, affect the probability of voting for these parties. Second, we move beyond economic evaluations and examine the effect of different types of performance evaluations. We start with a focus on economic evaluations, by far the most extensively tested notion of retrospective behavior. We then move to a more general concept of retrospective accountability, since we know today that the economy is not the only area in which the public may want to hold their representatives accountable (e.g. Singer 2011; De Vries and Giger 2014). This enables us to obtain a fully fledged picture on whether and how accountability mechanisms can affect political parties as voters’ most evident representatives (e.g. Strøm 2000).

The substantial application investigates data from a long-term panel of the German Longitudinal Election Study (GLES) providing a set of new measures that directly capture (dis)satisfaction with the performance for *all* parties represented in the parliament, the *Bundestag*. Germany is a good example of a multi-party system where opposition parties have a relatively strong influence on the policy-making process (Duch and Stevenson 2008). In addition, its history of coalition governments, where multiple parties compete for shares of policy-making responsibility, calls for a focus on the party level, rather than on the more abstract level of government versus opposition (Debus, Stegmaier, and Tosun 2014; Williams, Stegmaier, and Debus 2015).

The results indicate that the common assumption of retrospective evaluations having a unique or a stronger effect on government parties, when compared to the opposition, does not hold. Electoral accountability occurs at the party level with party size playing an important role in responsibility attribution to single parties, regardless of whether they were in government or in opposition. It is not only the prime minister or chancellor party which is affected the most by retrospective voting (Fisher and Hobolt 2010; Debus, Stegmaier, and Tosun 2014), but also the largest opposition party. We conclude by stating that future research should examine electoral accountability mechanisms at the party level, considering both government and opposition.

## 2. Retrospective voting at the party level

In a simple understanding of political representation, citizens constitute the principal that delegates authority in decision-making to political actors by voting for them in elections (Strøm 2000). In this context, electoral accountability is said to exist when citizens can retrospectively hold politicians accountable, and reward or punish them with their vote (e.g. Zelle 1995; Gidengil et al. 2001; Bélanger 2004; Dassonneville, Blais, and Dejaeghere 2015).^1^ The existing literature has examined the reward/punish mechanism by mainly focusing on the economic performance of government parties (e.g. Anderson 2007; Lewis-Beck, Nadeau, and Elias 2008). Empirical analyses regularly find that “when economic conditions are bad, citizens vote against the ruling party” (Lewis-Beck 1991, 2), even if the strength of economic voting differs substantially across studies and across settings (Duch and Stevenson 2008; Hobolt, Tilley, and Banducci 2013).

Very recently, however, the retrospective voting approach of examining the government performance collectively has come under increasing pressure (e.g. Fisher and Hobolt 2010; Debus, Stegmaier, and Tosun 2014). This approach is particularly problematic in countries with multi-party systems and coalition governments. For one, voters cannot vote for the government as a whole but only for one party of the coalition. Assuming identical influence of past performance evaluations on all coalition partners is an unrealistic restriction (e.g. Anderson 2000; Williams, Stegmaier, and Debus 2015). For another, in many contexts (e.g. federal systems and minority governments), the influence of parties out of government on policy-making is substantial where the responsibility attribution is less clear-cut between government and opposition parties and controversial policy decision can only be made in cooperation with the opposition (Strøm 1990; Powell and Whitten 1993; Lijphart 1999). It is therefore likely that voters are aware of and use this wider notion of responsibility when choosing parties in elections (Anderson 2000; Sanders and Carey 2003; Duch and Stevenson 2008; Duch and Falcó-Gimeno 2014). To generally evaluate parties, voters might simply take into consideration the obligations that parties are expected to fulfil in their role of opposition or government party (e.g. Hobolt and Tilley 2014). Hence, they might use different information when weighting the performance evaluation of government and opposition parties. While government parties might be judged more on their policy performance, the evaluation of opposition parties might be more likely driven by institutional aspects. These circumstances make it necessary to test the retrospective voting mechanism not only on the government but also at the party level, hence for both the government and the opposition.^2^

In a first step, we examine the impact of economic evaluations on all parties. In keeping with the literature, a positive (negative) economic evaluation should increase (decrease) the likelihood to vote for parties in government, and should have an opposite or no influence on vote choice for parties in opposition. In addition, and again in line with the existing literature, the effect of retrospective voting on the government parties’ vote should be stronger when citizens assign responsibility for the economic situation to the government. In a second step, we move from economic-centric evaluations to a general performance evaluation of parties. Here, we follow the most recent literature indicating that accountability extends to party performance across an array of policy areas and not only the economy (e.g. Fisher and Hobolt 2010; Singer 2011; De Vries and Giger 2014). Again, in line with a common approach, performance evaluations should have a unique or stronger impact on parties in government when compared to parties in opposition. If however, as we claim, retrospective voting works for parties both in government and in opposition, we should observe economic voting as well as general performance evaluations to have a different effect across parties and to matter for parties regardless of their government status.

## 3. Data, variables and methods

**Figure A1.**
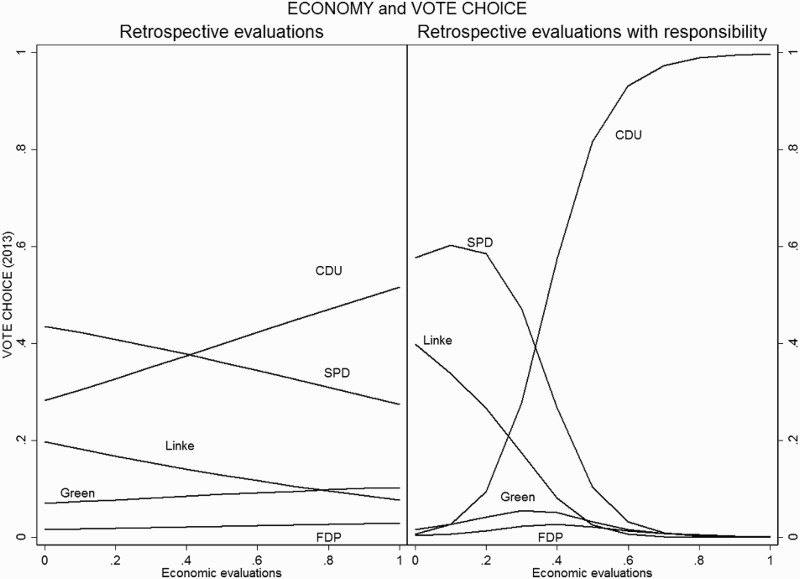
Retrospective economic voting for all parties (2009).

**Figure A2.**
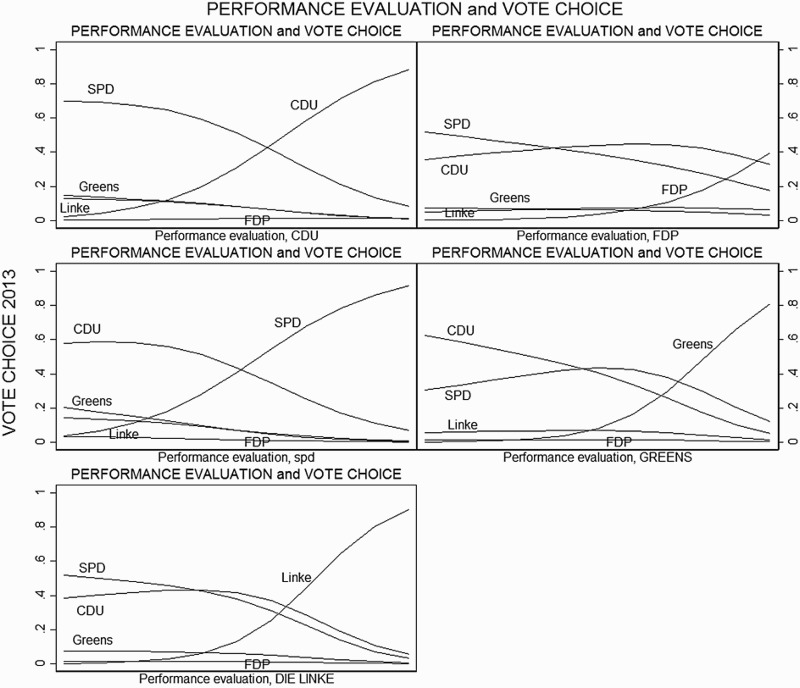
Retrospective evaluations and vote choice for all parties (2009).

The German Longitudinal Election Study, Component 8 (GLES) (Rattinger et al. 2014) (2009–2017) consists of several waves. It covers, among other things, the two federal elections of 2009 and 2013.^3^ Given that substantive conclusions are very similar for the two elections, in this paper, we only show the results for the 2013 elections and report results for the 2009 elections in the Appendix only (Figures A1 and A2).

Our dependent variable is party choice on Election Day. To measure the effect of retrospective evaluations on party choice, we apply multinomial logit models.^4^ The set of discrete alternatives consists of up to five parties that are regarded as being most likely to gain parliamentary representation: the two largest parties, the Christian Democratic Union/Christian Social Union (CDU/CSU) and the Social Democratic Party (SPD), and the smaller parties, the Free Democratic Party (FDP), the Greens and Die Linke (The Left).^5^

Turning to our independent variables, our main explanatory variable on economic performance is the economic situation as perceived by the voters: the variable measures whether the economy has worsened or improved using a scale from 1 (a lot worse) to 5 (improved a lot). A follow-up question asks respondents how strongly the policy of the federal government has affected the economic development, with options spanning from 1 (not at all) to 5 (very much), thus allowing respondents to ascribe levels of responsibility.

GLES also collects data on general performance for each of the parties represented in parliament using the following question: “*How* *satisfied or dissatisfied are you with the performance of X party in the government* [or *in the opposition*]*?*” The options available to respondents span a scale from −5 to +5 with higher scores representing a better policy performance. We recode all performance variables to range from 0 to 1, where 1 represents higher levels on that specific variable.

In all models, we include partisanship to isolate the effect of voters’ partisan affiliation on vote choice (e.g. Duch and Stevenson 2008; Tilley, Garry, and Bold 2008; Tilley and Hobolt 2011).^6^ We also control for satisfaction with democracy and several individual-level determinants such as age, gender and political interest.^7^

## 4. Empirical results

To test our expectations, we run three models of party choice for economic and general performance evaluations. Instead of presenting regression tables, we simply show the effect of each variable on the predicted probability of voting for a specific party; regression tables with the exact results are shown in the Appendix (Table A1).

**Figure 1.**
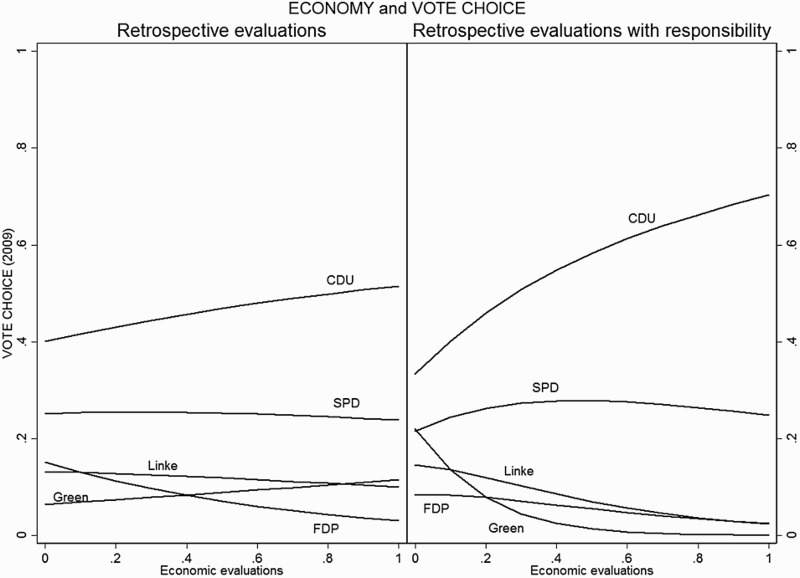
Retrospective economic voting for all parties.

Note: The graph shows the predicted probability of voting for a specific party based on Models 1 and 2, Table A1. Figures with confidence intervals can be found in the Online Supporting material.

Starting with the economy, the panel on the left of Figure 1 shows the effect of retrospective economic evaluations on party choice. The two government parties between 2009 and 2013 were the CDU/CSU and the FDP, the three opposition parties were the SPD, the Greens and Die Linke. We see that the CDU/CSU is the only party in government that was rewarded when respondents assessed the economy positively. The effect of positive economic evaluations on the junior coalition partner, the FDP, is very low to non-existent. For the parties in the opposition, the impact of positive economic retrospective evaluations is negative and only statistically significant for the largest party, the SPD, and the extreme party, Die Linke. The panel on the right shows the effect of retrospective economic evaluations on all parties; in this case, respondents regard the government as responsible for the actual situation of the economy in Germany. The effect on government is strong; however, the reward for positive economic evaluations is enjoyed only by the CDU/CSU. Again, only the SPD and Die Linke are affected in the opposition. In addition, it is important to note that the negative effect on opposition parties is only significant at the lower levels of economic evaluations (below 0.5 in the graph), and only when there is responsibility attribution (right panels). In sum, a positive economic evaluation benefits the largest government party and it has a negative effect on the largest party in the opposition; but it has a rather low effect on all other parties, regardless of whether they are in government or opposition.

**Figure 2.**
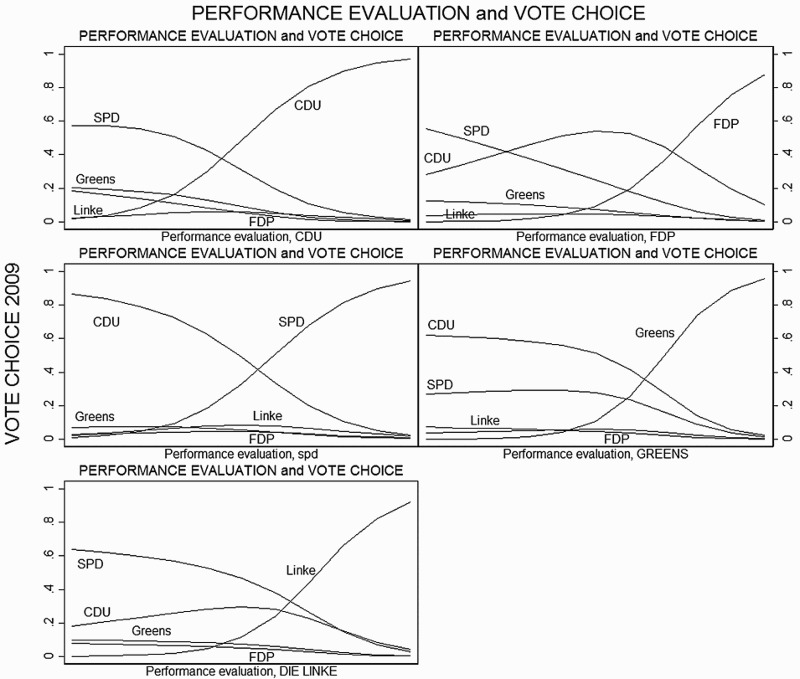
Retrospective general evaluations and vote choice for all parties.

Note: The graph shows the predicted probability of voting for a specific party based on Models 3, Table A1. Figures with confidence intervals can be found in the Online Supporting material.

In the next two steps, we examine performance evaluations beyond the economy. An overview across all panels of Figure 2 clearly indicates that the more citizens are (dis)satisfied with a party, the more likely they are (not) to vote for it. A closer look at each panel tells a more interesting story. On the one hand, concerning the two big parties, there is an almost parallel yet opposite effect of their performance on the probability of voting for the other party, respectively. On the other hand, the impact of the evaluations of the smaller parties on the probability of voting for one of the largest parties is quite low. Moving to the smaller parties, we see that the evaluations of the large parties exert a rather strong effect, pulling voters towards both large parties until the overall performance evaluations of that specific small party reach a point of moderate positive evaluations. At this point, the effect of retrospective evaluations on vote choice is quite large and just a little below the effects we observe for larger parties. While it seems that party size matters in establishing which performance is taken into account when citizens cast a ballot, we see that both large and small parties suffer from performance evaluations.^8^ The results are very similar if we look at the 2009 election (in the Appendix), which ended four years of a grand coalition government between the CDU/CSU and SPD that allowed both parties to control key cabinet posts for the economy (see Williams, Stegmaier, and Debus 2015). A comparison of the two elections indicates that past performance evaluations influence coalition partners differently, although the SPD in 2009 is affected much more by retrospective evaluations than the FDP in 2013 due to its more dominant role in the party system.

In sum, we find that the accountability mechanism does not appear to simply be a matter of government approval or rejection, but that it works mainly at the party level. In line with recent contributions (Debus, Stegmaier, and Tosun 2014; Duch and Falcó-Gimeno 2014; Williams, Stegmaier, and Debus 2015), our findings indicate that future research should examine electoral accountability mechanisms at the party level rather than at the more abstract level of government versus opposition. Above all, our findings show that both government and opposition parties can expect credit and blame for their past performance, and that both large and small parties are exposed to the workings of the retrospective mechanism. While these findings complement previous results for the European Parliament elections (Marsh 2009; Weber 2011), they are more meaningful as they show that opposition parties are also subject to retrospective voting in first-order elections.

## 5. Discussion and conclusion

This paper’s main aim was to update our knowledge on the retrospective voting mechanism by testing the notion that this mechanism works at the party level. This effort stems from two contentions. First, the retrospective voting mechanism, focusing on whether voters support or do not support the government, clearly works much better in single-party governments when compared to coalition governments. Second, the widespread disregard for parliamentary opposition in performance voting literature is largely unjustified when we move from plurality to consensual democracy and from two-party systems to multi-party systems. On the first issue, our results show that retrospective evaluations have a stronger effect on the senior coalition partner compared to the junior partner. This corroborates recent findings in the literature which indicate that the effect of past performance evaluations influences coalition partners differently (Fisher and Hobolt 2010; Debus, Stegmaier, and Tosun 2014; Williams, Stegmaier, and Debus 2015).

On the second issue, the often unspoken assumption in the literature that performance voting is based solely on voters’ evaluations of the government parties’ past performance does not find support in our study. In fact, higher expectations concerning the competence to deliver seem to become most noticeable among all parties, regardless of their government status. It shows that the mechanisms of electoral accountability function slightly more sophisticatedly than previously assumed.

Overall, our findings suggest that government parties should not be the only ones concerned about the blaming aspect, since all parties can suffer from it. Rather, it is the voters’ rationale to hold their previously supported party accountable and this is a key requirement for representative democracy (Strøm 2000). Given the significant implications of democratic accountability, it is clearly worth exploring whether our findings also hold in other countries. Therefore, we finally suggest that national election studies should adapt their measurement tools on electoral accountability to the party level for future analyses. In addition, while the focus on policy performance at the party level is well grounded and important, it would be worth establishing why people are satisfied with the performance of a given party regarding aspects such as the ability to compromise or vice versa of keeping closely to their positions.

## Supplementary Material

JEPOP-2016-0037.R2_ONLINE_APPENDIX.docx

## Notes

1 Voters may also decide to exit the system altogether (e.g. Weber 2011). Our data provide us with full information about vote choice. However, exit was rarely chosen, and for this reason, we do not focus on abstention in this paper. This decision also stems from the fact that our results must balance each other out and as support for one party goes up, support for some other party needs to go down.

2 Some research has also investigated retrospective voting at the level of the individual Member of Parliament (MP) finding that policy accountability of MPs is rather weak (e.g. Vivyan and Wagner 2012).

3 Sampling was based on multi-level random sampling of a population comprising all German residents of a minimum age of 16 years. Both the 2009 and 2013 surveys were face-to-face surveys with standardized questionnaires (CAPI). It is known that panelists are not a random sample out of the initial cross-section sample of respondents (Frankel and Hillygus 201 4; Warren and Halpern-Manners 2012). To address this issue, we run additional tests (e.g. Heckman probit selection models) to check whether the findings are sensitive to sample selection bias. Accounting for sample selection bias confirms our substantive conclusions.

4 A possible alternative to a multinomial logit model would be to run a conditional logit model for the general performance models and thereby include only one performance indicator for each party-choice. We, however, decided to run multinomial logit models for both the economic evaluation and the general performance to have the same dependent variable in both sets of models and provide a more straightforward comparison.

5 German voters cast two votes, a proportional vote to choose national parties and a plurality vote to choose local candidates. In this paper, we rely on the former because we are interested in the national performance of parties. Note, however, that switching to the candidate vote leads to similar albeit weaker conclusions. This would be in line with the idea that voter knowledge regarding candidates is lower than that of parties (e.g. Pattie and Johnston 2004).

6 Some have argued that people’s perceptions of policy performance are shaped by their political orientation – notably partisanship (Wlezien, Franklin, and Twiggs 1997; Duch, Palmer, and Anderson 2000; Evans and Andersen 2006) – others (e.g. Lewis-Beck, Nadeau, and Elias 2008) have questioned this. To account for a possible perceptual bias, that is, voters who feel close to a political party are likely to evaluate its performance more positively through a “partisan lens”, our supporting material shows additional models in which retrospective evaluations have been interacted with partisanship, and models in which party identification has been excluded all together. The findings support our substantive conclusions. In the Online Supporting Material, we also include Table S3 containing a correlation matrix of all evaluations and party identification variables.

7 Strategic voting is an important aspect of voting behavior within the German context (Pappi and Thurner 2002; Gschwend 2007). Using a 0–10 sympathy scale, we build a dummy variable that takes a value of 1 if the party voted for is the voter’s most preferred party, and 0 otherwise. Our results are robust to the addition of this variable.

8 Party size might capture other party characteristics such as the effect of mainstream versus that of niche parties.

## APPENDIX

**Table A1.** Models of vote choice: multinomial logit models (2013).

	SPD			FDP			Greens			Die Linke		
Reference: Vote CDU/CSU	(1)	(2)	(3)	(1)	(2)	(3)	(1)	(2)	(3)	(1)	(2)	(3)
Retrospective economy	−1.19+	7.45***		−0.01	3.84		−0.21	4.84*		−1.54+	8.13**	
	(0.63)	(2.09)		(0.93)	(3.31)		(0.85)	(2.21)		(0.87)	(2.66)	
Responsibility economy		7.87***			3.32			3.74+			8.18***	
		(1.83)			(2.95)			(2.04)			(2.26)	
Retrospective economy*		−12.95***			−5.72			−8.05*			−15.04***	
Responsibility economy		(3.09)			(4.70)			(3.51)			(3.89)	
General performance (CDU)			−5.77***			−2.33			−6.43***			−5.99***
			(1.03)			(1.50)			(1.23)			(1.23)
General performance (SPD)			−1.07			−0.99			0.46			−0.68
			(0.82)			(1.21)			(1.10)			(1.12)
General performance (FDP)			5.31***			5.42***			−1.70			−1.02
			(1.08)			(1.22)			(1.36)			(1.35)
General performance (Greens)			1.50			1.04			9.22***			0.91
			(0.97)			(1.11)			(1.48)			(1.27)
General performance (Linke)			0.76			0.30			−0.10			9.09***
			(0.81)			(0.97)			(1.00)			(1.35)
												
Party identification (CDU)	−1.90***	−1.75***	−1.32**	−0.28	−0.21	−0.76	−1.02+	−0.89	−0.34	−1.91***	−1.74**	−0.72
	(0.38)	(0.39)	(0.44)	(0.47)	(0.47)	(0.51)	(0.54)	(0.54)	(0.64)	(0.58)	(0.59)	(0.69)
Party identification (SPD)	3.86***	4.05***	2.89***	0.38	0.46	0.30	3.17***	3.22***	2.56***	2.55***	2.70***	2.41***
	(0.40)	(0.41)	(0.44)	(0.87)	(0.88)	(0.90)	(0.54)	(0.54)	(0.60)	(0.51)	(0.53)	(0.62)
Party identification (FDP)	0.63	0.75	1.01	3.20***	3.24***	2.62***	−34.37	−37.47	−17.76	0.25	0.40	2.02
	(0.82)	(0.83)	(1.02)	(0.73)	(0.73)	(0.79)	(4.1e + 07)	(2.0e + 08)	(13697.56)	(1.21)	(1.21)	(1.45)
Party identification (Greens)	1.80**	1.98**	1.07	−12.30	−11.79	−12.37	4.57***	4.61***	3.67***	0.99	1.11	1.21
	(0.61)	(0.63)	(0.69)	(585.58)	(471.39)	(715.45)	(0.63)	(0.65)	(0.74)	(0.92)	(0.94)	(1.05)
Party identification (Linke)	1.31+	1.36+	0.86	1.03	1.02	0.87	1.64+	1.62	1.57	4.67***	4.71***	3.91***
	(0.77)	(0.78)	(0.84)	(1.23)	(1.23)	(1.29)	(1.00)	(1.00)	(1.09)	(0.69)	(0.69)	(0.81)
Satisfaction with democracy	1.08+	0.90	0.36	−0.48	−0.50	−0.29	1.78*	1.81*	0.63	2.94***	2.82***	1.52+
	(0.60)	(0.60)	(0.70)	(0.83)	(0.83)	(0.96)	(0.76)	(0.77)	(0.89)	(0.73)	(0.74)	(0.91)
Age	0.00	0.00	0.01	0.01	0.02	0.01	−0.02+	−0.02+	−0.01	−0.00	0.00	0.01
	(0.01)	(0.01)	(0.01)	(0.01)	(0.01)	(0.01)	(0.01)	(0.01)	(0.01)	(0.01)	(0.01)	(0.01)
Female	−0.04	−0.13	−0.02	0.21	0.21	0.17	−0.28	−0.30	−0.24	0.01	−0.03	0.20
	(0.28)	(0.29)	(0.31)	(0.36)	(0.37)	(0.38)	(0.34)	(0.35)	(0.39)	(0.35)	(0.36)	(0.42)
Political interest	−0.67	−0.71	−0.38	−1.01	−1.11	−1.69+	−0.34	−0.42	0.23	−1.79*	−1.79*	−0.11
	(0.67)	(0.69)	(0.80)	(0.86)	(0.86)	(0.92)	(0.83)	(0.85)	(0.98)	(0.85)	(0.86)	(1.03)
Constant	−0.70	−6.00***	−1.33	−2.51*	−4.79*	−3.12*	−1.46	−3.91*	−2.06	−1.52	−7.06***	−4.27**
	(0.81)	(1.48)	(0.99)	(1.12)	(2.29)	(1.43)	(1.03)	(1.61)	(1.26)	(1.06)	(1.93)	(1.32)
*N*	851	851	851									
Pseudo-*R*^2^	0.45	0.46	0.56									
LL	−659.07	−646.16	−527.52									
AIC	1406.14	1396.31	1175.04									

Standard errors in parentheses:

+*p *< 0.1.

**p *< .05.

***p *< .01.

****p *< .001.
