# Supplementary material for: Retrospective voting and party support at elections: credit and blame for government and opposition
Source: J Elect Public Opin Parties. 2016 Oct 26;27(2):156–71. doi: 10.1080/17457289.2016.1243543 (PMC5407355; doi:10.1080/17457289.2016.1243543)
Supplement: JEPOP-2016-0037.R2_ONLINE_APPENDIX.docx [file fbep_a_1243543_sm3951.docx]

**ONLINE SUPPORTING MATERIAL**

As discussed in the paper, there is today significant evidence that partisan preferences influence retrospective evaluations. Several have in fact argued that peoples’ perceptions of policy performance are shaped by their political orientation – notably partisanship (Wlezien et al. 1997; Duch et al. 2000; Evans and Andersen 2006) – albeit others have questioned this (e.g., Lewis-Beck, Nadeau, and Elias 2008). To account for this perceptual bias, i.e., voters who feel close to a political party are likely to evaluate its performance more positively through a ‘partisan lens’, we show below the results of additional models in which retrospective evaluations have been interacted with partisanship, and models in which party identification has been excluded from the analyses all together. The results without party identification are shown in Table S1: a comparison between Table S1 (without a party identification variable ) and Table A1 (with party identification) shows that both the sign and the significance of the coefficients of the retrospective evaluations variables remain unchanged when we drop party identification from our empirical models, ultimately suggesting that the relationships of interest are robust to the inclusion or exclusion of the party identification variable. In addition, Table S2 presents models in which the variables measuring performance evaluations are interacted with variables measuring party identification. As the results indicate, while on a general level it seems that non identifiers tend to punish their own party to a lesser degree, the interaction terms between retrospective evaluations and party identification do not reach conventional level of statistical significance which suggests that perceptions of policy performance are not significantly shaped by people political orientation. Finally, Table S3 contains a correlation matrix of all evaluations and party identification variables. It shows that the correlation between party identification and performance evaluations are rather low and do not show any problematic patterns.

Table S1: Models of vote choice (without party identification): Multinomial logit models (2013)

| Reference: Vote CDU/CSU |  |  |  |  |  |  |  |  |  |  |  |  |
| --- | --- | --- | --- | --- | --- | --- | --- | --- | --- | --- | --- | --- |
|  | SPD | | | FDP | | | Greens | | | Die Linke | | |
|  | (1) | (2) | (3) | (1) | (2) | (3) | (1) | (2) | (3) | (1) | (2) | (3) |
| Retrospective economy | -1.91*** | 6.85*** |  | -0.59 | 3.35 |  | -1.22+ | 5.38** |  | -1.95** | 8.17*** |  |
|  | (0.45) | (1.63) |  | (0.85) | (2.97) |  | (0.63) | (2.02) |  | (0.59) | (2.03) |  |
| Responsibility economy |  | 6.39*** |  |  | 3.12 |  |  | 3.33+ |  |  | 7.26*** |  |
|  |  | (1.45) |  |  | (2.67) |  |  | (1.89) |  |  | (1.73) |  |
| Retrospective economy* |  | -13.75*** |  |  | -5.94 |  |  | -10.93*** |  |  | -15.89*** |  |
| Responsibility economy |  | (2.46) |  |  | (4.31) |  |  | (3.17) |  |  | (3.00) |  |
| General performance (CDU) |  |  | -9.75*** |  |  | -4.48*** |  |  | -9.62*** |  |  | -9.54*** |
|  |  |  | (0.89) |  |  | (1.33) |  |  | (1.06) |  |  | (1.03) |
| General performance (SPD) |  |  | -1.68** |  |  | 6.03*** |  |  | -1.03 |  |  | -1.23 |
|  |  |  | (0.63) |  |  | (1.13) |  |  | (0.90) |  |  | (0.90) |
| General performance (FDP) |  |  | 8.26*** |  |  | -1.35 |  |  | 0.24 |  |  | 0.03 |
|  |  |  | (0.94) |  |  | (1.14) |  |  | (1.16) |  |  | (1.13) |
| General performance (Greens) |  |  | 1.57* |  |  | 0.94 |  |  | 11.40*** |  |  | 0.51 |
|  |  |  | (0.80) |  |  | (1.06) |  |  | (1.34) |  |  | (1.02) |
| General performance (Linke) |  |  | 0.59 |  |  | 0.25 |  |  | -0.41 |  |  | 11.18*** |
|  |  |  | (0.65) |  |  | (0.93) |  |  | (0.83) |  |  | (1.17) |
|  |  |  |  |  |  |  |  |  |  |  |  |  |
| Satisfaction with democracy | 1.26** | 1.06* | 0.13 | -0.42 | -0.48 | -0.37 | 1.39* | 1.32* | 0.27 | 4.95*** | 4.73*** | 2.32** |
|  | (0.41) | (0.42) | (0.57) | (0.80) | (0.80) | (0.91) | (0.57) | (0.59) | (0.75) | (0.53) | (0.54) | (0.74) |
| Age | -0.01 | -0.00 | 0.01 | 0.01 | 0.01 | 0.00 | -0.03*** | -0.03*** | -0.02+ | -0.00 | 0.00 | 0.02 |
|  | (0.01) | (0.01) | (0.01) | (0.01) | (0.01) | (0.01) | (0.01) | (0.01) | (0.01) | (0.01) | (0.01) | (0.01) |
| Female | -0.21 | -0.15 | 0.00 | -0.20 | -0.19 | -0.28 | -0.08 | 0.08 | 0.03 | -0.28 | -0.19 | 0.04 |
|  | (0.18) | (0.18) | (0.24) | (0.33) | (0.33) | (0.34) | (0.25) | (0.26) | (0.32) | (0.24) | (0.25) | (0.34) |
| Political Interest | -0.85* | -0.94* | -0.44 | -1.36+ | -1.47+ | -1.82* | -1.28* | -1.38* | -0.55 | -2.32*** | -2.38*** | -0.73 |
|  | (0.42) | (0.43) | (0.61) | (0.78) | (0.79) | (0.84) | (0.60) | (0.61) | (0.80) | (0.57) | (0.58) | (0.81) |
| Constant | 1.07* | -3.14** | 0.56 | -1.29 | -3.36+ | -1.12 | 1.29+ | -0.87 | 0.25 | -1.01 | -5.87*** | -2.69* |
|  | (0.52) | (1.07) | (0.82) | (0.98) | (1.99) | (1.21) | (0.70) | (1.35) | (1.06) | (0.71) | (1.39) | (1.09) |
| *N* | 851 | 851 | 851 |  |  |  |  |  |  |  |  |  |
| Pseudo-R2 | 0.18 | 0.21 | 0.43 |  |  |  |  |  |  |  |  |  |
| LL | -1105.85 | -1069.14 | -683.49 |  |  |  |  |  |  |  |  |  |
| AIC | 2259.69 | 2202.29 | 1446.99 |  |  |  |  |  |  |  |  |  |

Standard errors in parentheses: + p<0.1, * p<.05, ** p<.01, *** p<.001

Table S2: Models of vote choice (moderate by party identification): Multinomial logit models (2013)

| Reference: Vote CDU/CSU |  |  |  |  |  |  |  |  |  |  |  |  |
| --- | --- | --- | --- | --- | --- | --- | --- | --- | --- | --- | --- | --- |
|  | SPD |  |  | FDP |  |  | Greens |  |  | Die Linke |  |  |
|  | (1) | (2) | (3) | (1) | (2) | (3) | (1) | (2) | (3) | (1) | (2) | (3) |
| Retrospective economy | -1.10 | 9.63*** |  | -0.95 | 2.40 |  | 0.62 | 7.56* |  | -0.17 | 11.84*** |  |
|  | (1.20) | (2.89) |  | (2.01) | (4.25) |  | (2.02) | (3.68) |  | (1.66) | (3.32) |  |
| Responsibility economy |  | 10.35*** |  |  | 3.38 |  |  | 6.74* |  |  | 10.35*** |  |
|  |  | (2.26) |  |  | (3.18) |  |  | (2.63) |  |  | (2.61) |  |
| Retrospective economy* |  | -14.78*** |  |  | -5.20 |  |  | -10.03* |  |  | -18.40*** |  |
| Responsibility economy |  | (3.91) |  |  | (5.92) |  |  | (4.89) |  |  | (4.61) |  |
| General performance (CDU) |  |  | -6.71*** |  |  | 0.05 |  |  | -7.58*** |  |  | -7.14*** |
|  |  |  | (1.24) |  |  | (2.27) |  |  | (1.45) |  |  | (1.43) |
| General performance (SPD) |  |  | 6.21*** |  |  | -0.95 |  |  | 0.39 |  |  | -1.78 |
|  |  |  | (1.33) |  |  | (1.27) |  |  | (1.73) |  |  | (1.63) |
| General performance (FDP) |  |  | -1.49+ |  |  | 5.80*** |  |  | 0.14 |  |  | -1.02 |
|  |  |  | (0.83) |  |  | (1.34) |  |  | (1.12) |  |  | (1.12) |
| General performance (Greens) |  |  | 1.46 |  |  | 1.07 |  |  | 9.63*** |  |  | 1.08 |
|  |  |  | (1.00) |  |  | (1.14) |  |  | (1.66) |  |  | (1.30) |
| General performance (Linke) |  |  | 0.52 |  |  | 0.30 |  |  | -0.22 |  |  | 8.64*** |
|  |  |  | (0.81) |  |  | (1.00) |  |  | (1.02) |  |  | (1.49) |
|  |  |  |  |  |  |  |  |  |  |  |  |  |
|  |  |  |  |  |  |  |  |  |  |  |  |  |
| Party identification (CDU) | -3.66** | -3.53* | -4.57* | -0.98 | -1.20 | 2.41 | 0.25 | 0.32 | -1.35 | 1.19 | 0.29 | -4.18+ |
|  | (1.32) | (1.40) | (2.03) | (1.39) | (1.46) | (2.13) | (1.67) | (1.72) | (1.87) | (1.41) | (1.52) | (2.31) |
| Party identification (CDU)* | 2.80 | 2.50 |  | 1.22 | 2.85 |  | -2.16 | -1.43 |  | -6.55* | -6.66 |  |
| Retrospective economy | (2.02) | (2.90) |  | (2.32) | (3.30) |  | (2.73) | (3.79) |  | (2.93) | (5.26) |  |
| Party identification (SPD) | 3.45** | 4.82*** | 4.96*** | 0.47 | 0.60 | 0.22 | 3.05+ | 3.86* | 5.63*** | 2.46+ | 3.33* | 2.25 |
|  | (1.12) | (1.25) | (1.49) | (2.34) | (2.39) | (3.26) | (1.62) | (1.72) | (1.70) | (1.42) | (1.53) | (1.80) |
| Party identification (SPD)* | 0.72 | 3.33 |  | -0.22 | 3.49 |  | 0.24 | 3.62 |  | 0.12 | 1.83 |  |
| Retrospective economy | (1.94) | (3.39) |  | (4.21) | (6.53) |  | (2.68) | (4.04) |  | (2.45) | (3.69) |  |
| Party identification (FDP) | 3.44 | 4.57 | -3.46 | 1.80 | 1.85 | 2.05 | -11.01 | -9.10 | -3.82 | 1.20 | -9.79 | 11.70 |
|  | (3.27) | (3.27) | (3.05) | (2.77) | (2.95) | (2.54) | (274.15) | (990.87) | (479.77) | (4.29) | (528.77) | (613.17) |
| Party identification (FDP)* | -5.90 | -2.17 |  | 2.51 | -3.15 |  | -14.84 | 52.65 |  | -1.79 | 115.34 |  |
| Retrospective economy | (6.44) | (9.28) |  | (4.75) | (7.26) |  | (820.33) | (216.42) |  | (7.95) | (963.64) |  |
| Party identification (Greens) | 2.89 | 6.71* | 2.32 | -19.71 | -14.46 | -14.60 | 6.96** | 10.05*** | 6.29* | 4.77+ | 8.11* | 5.61 |
|  | (2.38) | (2.80) | (2.66) | (198.36) | (127.11) | (116.83) | (2.46) | (2.86) | (2.66) | (2.79) | (3.35) | (4.06) |
| Party identification (Greens)* | -1.65 | 2.93 |  | 7.18 | 1.81 |  | -3.97 | 1.19 |  | -7.27 | -4.35 |  |
| Retrospective economy | (3.70) | (4.95) |  | (272.37) | (239.92) |  | (3.78) | (5.04) |  | (5.13) | (7.21) |  |
| Party identification (Linke) | 5.31+ | 6.54* | -3.82 | 3.86 | 4.89 | 2.18 | -1.00 | 0.06 | 0.53 | 7.54** | 8.47** | 2.34 |
|  | (2.82) | (3.10) | (3.16) | (3.99) | (4.32) | (5.04) | (4.80) | (5.41) | (3.81) | (2.64) | (2.88) | (2.56) |
| Party identification (Linke)* | -7.50 | -8.95 |  | -4.82 | -3.37 |  | 3.70 | 6.61 |  | -4.88 | -6.15 |  |
| Retrospective economy | (4.74) | (6.57) |  | (6.92) | (8.70) |  | (6.56) | (8.34) |  | (4.00) | (5.10) |  |
|  |  |  |  |  |  |  |  |  |  |  |  |  |
| Party identification (CDU)* |  | 0.34 |  |  | -1.85 |  |  | -1.10 |  |  | 3.46 |  |
| Retrospective*Responsibility economy |  | (3.26) |  |  | (3.86) |  |  | (4.62) |  |  | (6.88) |  |
| Party identification (SPD)* |  | -7.12+ |  |  | -6.07 |  |  | -7.19 |  |  | -4.13 |  |
| Retrospective*Responsibility economy |  | (4.01) |  |  | (8.23) |  |  | (4.82) |  |  | (4.59) |  |
| Party identification (FDP) * |  | -8.98 |  |  | 9.41 |  |  | -131.33 |  |  | -250.30 |  |
| Retrospective*Responsibility economy |  | (15.46) |  |  | (9.99) |  |  | (542.12) |  |  | (268.00) |  |
| Party identification (Greens)* |  | -17.66* |  |  | -5.07 |  |  | -16.86* |  |  | -13.54 |  |
| Retrospective*Responsibility economy |  | (6.98) |  |  | (294.84) |  |  | (6.96) |  |  | (10.51) |  |
| Party identification (Linke)* |  | -0.81 |  |  | -5.69 |  |  | -9.30 |  |  | -0.07 |  |
| Retrospective*Responsibility economy |  | (8.48) |  |  | (10.81) |  |  | (8.36) |  |  | (6.26) |  |
|  |  |  |  |  |  |  |  |  |  |  |  |  |
| Party identification (CDU)* |  |  | 4.49+ |  |  | -4.19 |  |  | 1.43 |  |  | 5.32+ |
| General performance (CDU) |  |  | (2.64) |  |  | (2.74) |  |  | (2.61) |  |  | (3.08) |
| Party identification (SPD) |  |  | -3.47 |  |  | -0.13 |  |  | -5.69* |  |  | 0.73 |
| General performance (SPD) |  |  | (2.40) |  |  | (4.89) |  |  | (2.88) |  |  | (2.95) |
| Party identification (FDP)* |  |  | 9.47 |  |  | 1.98 |  |  | -58.76 |  |  | -108.20 |
| General performance (FDP) |  |  | (5.83) |  |  | (5.09) |  |  | (265.43) |  |  | (724.40) |
| Party identification (Greens)* |  |  | -2.24 |  |  | -1.35 |  |  | -4.67 |  |  | -7.33 |
| General performance (Greens) |  |  | (4.44) |  |  | (221.62) |  |  | (4.42) |  |  | (7.00) |
| Party identification (Linke)* |  |  | 8.09 |  |  | -2.30 |  |  | 2.62 |  |  | 3.80 |
| General performance (Linke) |  |  | (5.44) |  |  | (9.90) |  |  | (6.69) |  |  | (4.90) |
| Satisfaction with democracy | 1.00+ | 0.87 | 0.50 | -0.43 | -0.78 | -0.18 | 1.75* | 1.80* | 0.74 | 2.98*** | 3.15*** | 1.62+ |
|  | (0.60) | (0.62) | (0.72) | (0.83) | (0.85) | (0.96) | (0.76) | (0.78) | (0.92) | (0.74) | (0.78) | (0.95) |
| Age | 0.00 | 0.01 | 0.01 | 0.02 | 0.01 | 0.01 | -0.02+ | -0.02 | -0.01 | -0.00 | 0.00 | 0.01 |
|  | (0.01) | (0.01) | (0.01) | (0.01) | (0.01) | (0.01) | (0.01) | (0.01) | (0.01) | (0.01) | (0.01) | (0.01) |
| Female | -0.07 | -0.09 | 0.08 | 0.25 | 0.30 | 0.17 | -0.33 | -0.30 | -0.21 | -0.02 | -0.07 | 0.30 |
|  | (0.28) | (0.29) | (0.32) | (0.36) | (0.37) | (0.38) | (0.34) | (0.36) | (0.40) | (0.36) | (0.38) | (0.43) |
| Political Interest | -0.65 | -0.70 | -0.22 | -1.08 | -1.22 | -1.83+ | -0.31 | -0.40 | 0.31 | -1.95* | -1.83* | -0.07 |
|  | (0.68) | (0.70) | (0.81) | (0.86) | (0.88) | (0.93) | (0.84) | (0.87) | (1.00) | (0.86) | (0.89) | (1.05) |
| Constant | -0.65 | -8.33*** | -1.26 | -2.07 | -4.07 | -4.97* | -1.90 | -6.82** | -2.59+ | -2.18 | -9.56*** | -3.29* |
|  | (0.98) | (1.97) | (1.15) | (1.47) | (2.62) | (2.03) | (1.52) | (2.47) | (1.53) | (1.33) | (2.42) | (1.55) |
| *N* | 851 | 851 | 851 |  |  |  |  |  |  |  |  |  |
| Pseudo-R2 | 0.46 | 0.48 | 0.57 |  |  |  |  |  |  |  |  |  |
| LL | -647.85 | -620.04 | -512.24 |  |  |  |  |  |  |  |  |  |
| AIC | 1423.71 | 1424.08 | 1184.48 |  |  |  |  |  |  |  |  |  |

Standard errors in parentheses: + p<0.1, * p<.05, ** p<.01, *** p<.001

Table S3: Correlation coefficients of all evaluation and party identification variables (2013)

|  |  |  | PID | | | | | General performance | | | |
| --- | --- | --- | --- | --- | --- | --- | --- | --- | --- | --- | --- |
|  | Retrospective economy | Responsibility | CDU | SPD | FDP | G | Die Linke | CDU | SPD | FDP | G |
| Responsibility | -0,134 | 1 |  |  |  |  |  |  |  |  |  |
| PID |  |  |  |  |  |  |  |  |  |  |  |
| CDU | 0,189 | 0,151 | 1 |  |  |  |  |  |  |  |  |
| SPD | -0,107 | -0,114 | -0,483 | 1 |  |  |  |  |  |  |  |
| FDP | -0,010 | -0,018 | -0,115 | -0,102 | 1 |  |  |  |  |  |  |
| G | -0,019 | -0,119 | -0,212 | -0,188 | -0,045 | 1 |  |  |  |  |  |
| Die Linke | -0,097 | 0,008 | -0,260 | -0,231 | -0,055 | -0,101 | 1 |  |  |  |  |
| General performance |  |  |  |  |  |  |  |  |  |  |  |
| CDU | 0,275 | 0,098 | **0,581** | -0,320 | 0,012 | -0,137 | -0,310 | 1 |  |  |  |
| SPD | 0,029 | -0,026 | -0,157 | **0,318** | -0,097 | 0,073 | -0,097 | 0,034 | 1 |  |  |
| FDP | 0,151 | 0,153 | 0,456 | -0,251 | **0,109** | -0,167 | -0,200 | 0,552 | 0,063 | 1 |  |
| G | 0,002 | -0,092 | -0,200 | 0,198 | -0,087 | **0,264** | -0,027 | -0,102 | 0,509 | -0,089 | 1 |
| Die Linke | -0,088 | 0,028 | -0,285 | 0,094 | -0,102 | 0,037 | **0,385** | -0,270 | 0,294 | -0,156 | 0,408 |

Table S4: Descriptive statistics of key independent variables (2013)

| Variable | Mean | Std. Dev. | Min. | Max. | N |
| --- | --- | --- | --- | --- | --- |
| Retrospective economy | 0.563 | 0.199 | 0 | 1 | 851 |
| Responsibility economy | 0.636 | 0.207 | 0 | 1 | 851 |
| General performance (CDU) | 0.586 | 0.256 | 0 | 1 | 851 |
| General performance (SPD) | 0.345 | 0.250 | 0 | 1 | 851 |
| General performance (FDP) | 0.518 | 0.197 | 0 | 1 | 851 |
| General performance (Greens) | 0.472 | 0.208 | 0 | 1 | 851 |
| General performance (Linke) | 0.411 | 0.246 | 0 | 1 | 851 |

Figure S1: Retrospective economic voting for all parties (with 95% confidence interval) (2013)


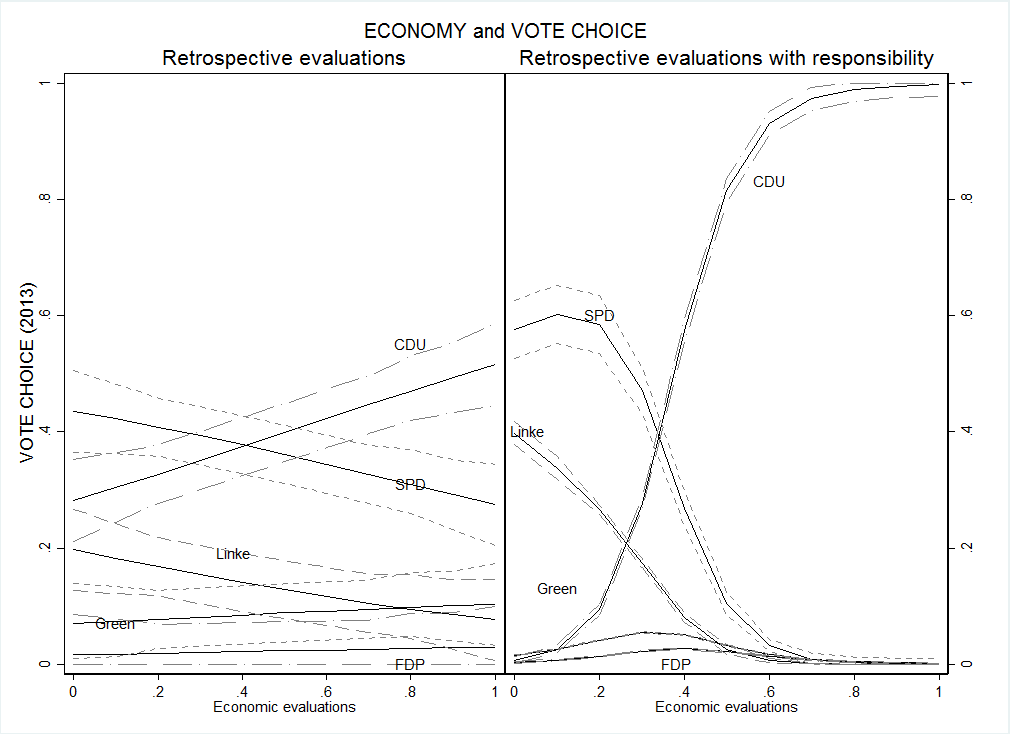


Note: based on Models 1 and 2, Table A1.

Figure S2: Retrospective general evaluations and vote choice for all parties (with 95% confidence interval) (2013)


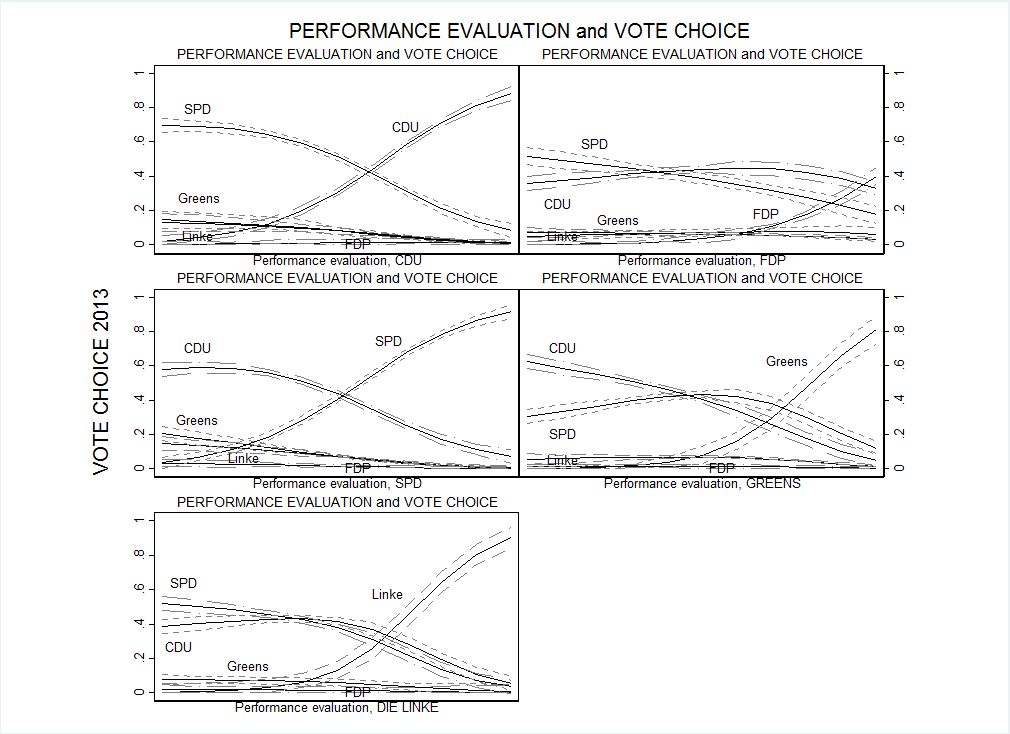


Note: based on Models 3, Table A1.
